# Functional subcellular distribution of β_1_- and β_2_-adrenergic receptors in rat ventricular cardiac myocytes

**DOI:** 10.1002/phy2.38

**Published:** 2013-08-22

**Authors:** Caroline Cros, Fabien Brette

**Affiliations:** Faculty of Life Sciences, The University of ManchesterManchester, M13 9NT, U.K

**Keywords:** Calcium, cardiac myocyte, t-tubules, β_1_-adrenergic receptors, β_2_-adrenergic receptors

## Abstract

β-adrenergic stimulation is a key regulator of cardiac function. The localization of major cardiac adrenergic receptors (β_1_ and β_2_) has been investigated using biochemical and biophysical approaches and has led to contradictory results. This study investigates the functional subcellular localization of β_1_- and β_2_-adrenergic receptors in rat ventricular myocytes using a physiological approach. Ventricular myocytes were isolated from the hearts of rat and detubulated using formamide. Physiological cardiac function was measured as Ca^2+^ transient using Fura-2-AM and cell shortening. Selective activation of β_1_- and β_2_-adrenergic receptors was induced with isoproterenol (0.1 μmol/L) and ICI-118,551 (0.1 μmol/L); and with salbutamol (10 μmol/L) and atenolol (1 μmol/L), respectively. β_1_- and β_2_-adrenergic stimulations induced a significant increase in Ca^2+^ transient amplitude and cell shortening in intact rat ventricular myocytes (i.e., surface sarcolemma and t-tubules) and in detubulated cells (depleted from t-tubules, surface sarcolemma only). Both β_1_- and β_2_-adrenergic receptors stimulation caused a greater effect on Ca^2+^ transient and cell shortening in detubulated myocytes than in control myocytes. Quantitative analysis indicates that β_1_-adrenergic stimulation is ∼3 times more effective at surface sarcolemma compared to t-tubules, whereas β_2_- adrenergic stimulation occurs almost exclusively at surface sarcolemma (∼100 times more effective). These physiological data demonstrate that in rat ventricular myocytes, β_1_-adrenergic receptors are functionally present at surface sarcolemma and t-tubules, while β_2_-adrenergic receptors stimulation occurs only at surface sarcolemma of cardiac cells.

## Introduction

In cardiac myocytes, sympathetic stimulation by β-adrenergic receptors activation is a key regulator of physiological function by controlling heart rate (chronotropic effect), strength of cardiac contraction (inotropic effect), and speed of cardiac relaxation (lusitropic effect, see Bers 2002 for review). These effects result from activation of the cAMP-dependent protein kinase (PKA) pathway via cAMP production and subsequent phosphorylation of key proteins in the excitation-contraction coupling (ECC) pathway, including the following: (i) L-type Ca^2+^ channels, increasing calcium current; (ii) ryanodine receptors, increasing their sensitivity to trigger Ca^2+^ release; and (iii) the regulatory protein phospholamban, increasing sarcoplasmic reticulum Ca^2+^ uptake via sarcoplasmic reticulum ATPase. The net effect of these changes is to increase the amplitude of the Ca^2+^ transient, thus increasing the strength of contraction (see Bers 2002 for review).

In the heart, several β-adrenergic receptors are expressed. β_1_-adrenergic receptors play a dominant role in increasing chronotropy and inotropy, whereas β_2_-adrenergic receptors produce only modest chronotorpic effects. A third type of β-adrenergic receptors (β_3_) is also expressed in the heart, but plays a minor functional role in heart (Xiang 2011). The concept of spatiotemporal regulation of cAMP and PKA activity is essential for fine tuning of signal transduction, but it is still not clear how the spatial response differs between β_1_- and β_2_-adrenergic receptors. Indeed, adrenergic stimulation induces distinct spatial and temporal responses in ventricular myocytes: β_1_-adrenergic receptor stimulation causes a diffuse cAMP response (global); while β_2_-adrenergic receptors stimulation is locally confined (Xiang 2011). Despite being a diffusible small molecule, cAMP diffusion is limited because of degradation by specific phosphodiesterases (Fischmeister et al. 2006), which can differentially regulate cAMP production and diffusion by β_1_- and β_2_-adrenergic receptors stimulation, thus cardiac function. In addition, it has been shown that β_2_-adrenergic receptors are also coupled to a G_*i*_-protein that counters the effects of the G_*s*_-protein signalling (Kuschel et al. 1999). Finally, the spatial localization of β-adrenergic receptors can play a critical role in the physiological response because of the membrane's unique membrane structure, namely the extensive network of t-tubules (large sarcolemmal invaginations at every *Z* lines) essential for synchronizing Ca^2+^ release within the myocyte (Brette and Orchard 2003) and the caveolae (small sarcolemmal invaginations discretely distributed along both surface sarcolemma and t-tubules), which are implicated in macromolecular signalling complexes (Harvey and Calaghan 2012). Recent compelling studies have shown that caveolae contribute to the compartmentalization of β_2_-adrenergic receptors signal in cardiac myocytes (e.g., Calaghan and White 2006). The subcellular distribution of β-adrenergic receptors within ventricular myocytes, and hence their possible role in compartmentalization, has been poorly investigated, particularly at the physiological level. Because the t-tubules are the main site of ECC and underlie synchronous Ca^2+^ release, this subcellular localization is critical in advancing our understanding of ECC modulation.

Biochemical and biophysical characterization have led to conflicting results. Immunohistochemical data have suggested that β_1_- and β_2_-adrenergic receptors are present at the surface membrane and the t-tubules (Zhou et al. 2000). Although these studies provide valuable information, quantification of protein distribution (surface sarcolemma vs. t-tubules) from immunostaining data is difficult. Using radioligand binding, it has been determined that β_1_-adrenergic receptors density is almost two times larger in surface sarcolemma than t-tubules (∼65% vs. ∼35% distribution, respectively), whereas β_2_-adrenergic receptors density is evenly distributed between surface sarcolemma and t-tubules (He et al. 2005). Other quantitative data have been obtained by the recent advance in live-cell imaging. By combining the smart patch-clamp technique and fluorescence resonance energy transfer (FRET)-based sensor (Epac2-camps to monitor cAMP production), it was found that β_1_-adrenergic receptors are evenly distributed between surface sarcolemma and t-tubules, while β_2_-adrenergic receptors are exclusively located at the t-tubules of ventricular myocytes (Nikolaev et al. 2010).

The aim of this study is to address the functional subcellular localization of β_1_- and β_2_-adrenergic receptors using a physiological approach. We have measured changes in Ca^2+^ transient and cell shortening (i.e., physiological functional response in cardiac myocytes) after selective β_1_- and β_2_-adrenergic receptors stimulation in intact rat ventricular myocytes and used acute detubulation, which enables us to determine the functional localization of proteins in ventricular myocytes; surface sarcolemma versus t-tubules (Brette et al. 2002; Pasek et al. 2008).

## Material and Methods

### Isolation and detubulation of rat ventricular myocytes

Myocytes were isolated from ventricles of Wistar rat hearts using a standard enzymatic dissociation protocol (Trafford et al. 1997). Detubulation was induced by osmotic shock as described previously (Brette et al. 2002). All experiments were performed at room temperature (∼22°C). All procedures were performed in compliance with the UK Home Office Animals (Scientific Procedures) Act 1986.

### Solutions

The physiological saline solution (Tyrode solution) contained (in mmol/L): 137 NaCl, 5 KCl, 1 CaCl_2_, 1 MgCl_2_, 10 glucose and 20 HEPES (pH 7.4 with NaOH). To selectively stimulate β_1_-adrenergic receptors, cells were perfused with isoproterenol (ISO, 0.1 μmol/L) and the β_2_-adrenergic receptors antagonist ICI 118,551 (ICI, 0.1 μmol/L). To selectively stimulate β_2_-adrenergic receptors, cells were perfused with salbutamol (10 μmol/L) and the β_1_-adrenergic receptors antagonist atenolol (1 μmol/L) (Calaghan and White 2006). All chemicals and drugs were purchased from Sigma (St. Louis, MO).

### Recordings of Ca^2+^ transients

Rat ventricular cells were loaded with the Ca^2^^+^ -sensitive fluorescent indicator Fura 2-AM (5 μmol/L; Molecular Probes, Grand Island, NY) for 10 min at room temperature. Cells were electrically field stimulated at 0.33 Hz with a pair of platinum electrodes. The ratio of fluorescence emitted at 510 nm in response to alternate excitation with 340 and 380 nm light was used as an index of intracellular Ca^2+^.

### Cell shortening measurements

Myocyte contractions were imaged with a video-edge detection system (Crescent Electronics, Sandy, UT) as previously described (Steadman et al. 1988). Cell contraction was induced by electrical field stimulation at 0.33 Hz with a pair of platinum electrodes. Images of cell contraction were captured with a charged-coupled device camera and displayed on a video monitor (Bosch, Frankfurt am Main, Germany).

### Data analysis

Analysis was performed using Clampfit (Molecular Devices, Sunnyvale, CA) and Origin (Microcal, Northampton, MA) software and by averaging 10 signals at steady state.

### Statistics

Data are presented as mean ± SEM. Two-way analysis of variance (ANOVA) followed by Tukey post hoc analysis was used to compare data from control and detubulated cells and to test the effect of β-adrenergic stimulation. One-way ANOVA was used to compare the percentage of increase of the amplitude of the Ca^2+^ transient during specific β-adrenergic stimulations between control and detubulated cells. T-tubules value was calculated (see results) therefore no statistical analysis can be performed. *P* < 0.05 was taken as significant.

## Results

To reconcile the conflicting biochemical and biophysical data regarding β_1_- and β_2_-adrenergic receptors distribution in ventricular myocytes, we conducted our experiments in a physiologically relevant system by investigating the effect of specific β agonists in control (i.e., surface sarcolemma and t-tubules stimulation) and detubulated (only surface sarcolemma stimulation) myocytes upon cardiac cell function.

### Effect of β_1_-adrenergic stimulation on cardiac function

**Figure 1:**
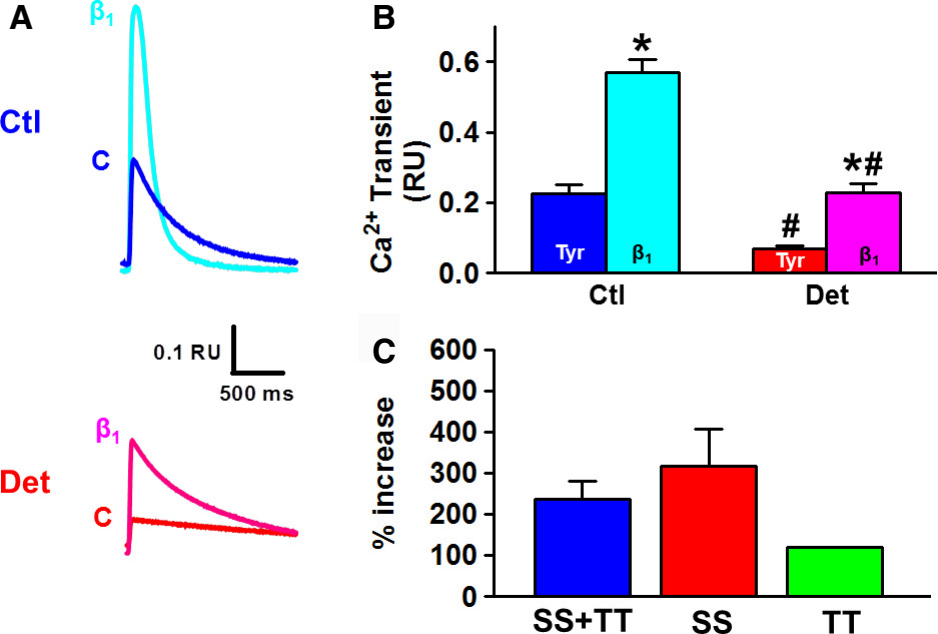
Effect of selective β_1_-adrenergic stimulation on Ca^2+^ transient of control and detubulated rat ventricular myocytes. (A) Ca^2+^ transient in representative control (Ctl) and detubulated (Det) myocytes before (bleu and red traces) and after (cyan and pink traces) selective β_1_-adrenergic stimulation. (B) Mean ± SEM of Ca^2+^ transient amplitude under control condition (Tyr) and after β_1_-adrenergic stimulation (β_1_). Data are from 29 control and 15 detubulated cells. (C) Mean ± SEM percentage increase in the Ca^2+^ transient after β_1_-stimulation in control myocytes (surface sarcolemma + t-tubules; SS + TT) and detubulated myocytes (SS, surface sarcolemma). From these values, we have calculated the percentage of increase at the TT (see text for details). #*P* < 0.05 between control and detubulated cells; *P < 0.05 between Tyrode and β_1_-adrenergic stimulation.

Figure 1A shows representative Ca^2+^ transients recorded from control (top) and detubulated (bottom) rat ventricular myocytes before (blue and red traces, respectively) and during perfusion with specific β_1_-adrenergic stimulation (0.1 μmol/L ISO and 0.1 μmol/L ICI; cyan and pink traces, respectively). The mean data presented in Figure 1B show that specific β_1_-adrenergic stimulation induced a large significant increase in the Ca^2+^ transient amplitude in both populations of cells (0.569 ± 0.037 RU [Ratio Unit], *n* = 29 control cells vs. 0.227 ± 0.027 RU *n* = 15, detubulated cells, two-way ANOVA, *P* < 0.05). As previously described (e.g., [Brette et al. 2004]), in the absence of β_1_-adrenergic stimulation, detubulation significantly reduced the amplitude of the Ca^2+^ transient (0.226 ± 0.024 RU in control cells, vs. 0.069 ± 0.008 RU in detubulated cells, (two-way ANOVA, *P* < 0.05). In addition, the time to decay to 50% of Ca^2+^ transient was significantly increased after detubulation (two-way ANOVA, *P* < 0.05, 380 ± 18 msec vs. 835 ± 55 msec, respectively), as previously described (Brette et al. 2002, 2004). Interestingly, application of specific β_1_-adrenergic stimulation caused a significant increase in the Ca^2+^ transient amplitude that appears to be larger in detubulated myocytes, although it does not reach significance (237 ± 43% in control vs. 317 ± 89% in detubulated cells, one-way ANOVA, *P >* 0.05, Fig. 1C). This confirms that β-adrenergic stimulation is effective in detubulated myocytes (using ISO only, see [Brette et al. 2004; Smyrnias et al. 2010]). In addition, the time to decay to 50% of Ca transient was significantly decreased after β_1_-adrenergic stimulation in both cell types (two-way ANOVA, *P* < 0.05, 177 ± 9 msec vs. 444 ± 55 msec, respectively), as previously described (Brette et al. 2004).

We calculated the increase in Ca^2+^ transient amplitude at the t-tubules by subtracting the Ca^2+^ transient value from control myocytes (i.e., surface sarcolemma and t-tubules) to detubulated myocytes value (surface sarcolemma only), as previously described (Despa et al. 2003; Brette and Orchard 2006; Pasek et al. 2008). We estimated that the percentage increase in Ca^2+^ transient at the t-tubules membrane was 119%, suggesting that β_1_-adrenergic stimulation is **˜**3 times more affective at surface sarcolemma than at t-tubules (Table 1). This further indicates a similar role of surface sarcolemma and t-tubules to the increase in Ca^2+^ transient (50% and 50%, Table 1).

**Table 1:** Distribution of mean (±SEM) ΔCa^2+^ transient and percentage increase during specific β_1_- and β_2_-adrenergic receptors stimulation in surface sarcolemma (SS) and t-tubules (TT)

	β_1_-AR stimulation		β_2_-AR stimulation	
		
	ΔCa^2+^ transient (RU)	% increase	ΔCa^2+^ transient (RU)	% increase
SS + TT (control cells)	0.343 ± 0.013	237 ± 43	0.036 ± 0.010	25 ± 4
SS (detubulated cells)	0.158 ± 0.019	317 ± 89	0.035 ± 0.004	83 ± 19
TT (calculated)	0.185	119	0.001	0.8
% functional response at TT	54	50	2.8	3.2
Stimulation efficiency (SS/TT)	0.85	2.66	35	104

Data are from 29 control and 15 detubulated cells for β_1_-adrenergic receptor stimulation and 41 control and 20 detubulated cells for β_2_-adrenergic receptor stimulation. TT was calculated as [(SS + TT) − SS]. See text for details about calculation and quantification.

**Figure 2:**
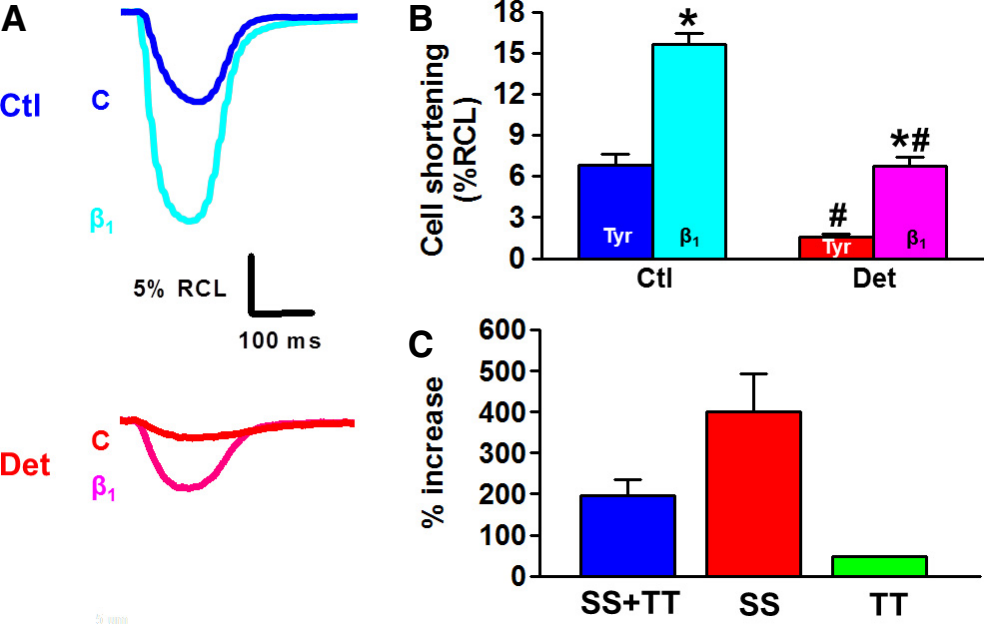
Effect of selective β_1_-adrenergic stimulation on cell shortening of control and detubulated rat ventricular myocytes. (A) Cell shortening in representative control (Ctl) and detubulated (Det) myocytes before (blue and red traces) and after (cyan and pink traces) selective β_1_-adrenergic stimulation. (B) Mean ± SEM of cell shortening expressed as a percentage of resting cell length (RCL) under control condition (Tyr) and after β_1_-adrenergic stimulation (β_1_). Data are from 22 control and 13 detubulated cells. (C) Mean ± SEM of the percentage increase in cell shortening after β_1_-stimulation in control myocytes (surface sarcolemma+t-tubules; SS + TT) and detubulated myocytes (SS, surface sarcolemma). From these values, we have calculated the percentage of increase at the t-tubules (TT, see text for details). #*P* < 0.05 between control and detubulated cells; *P < 0.05 between Tyrode and β_1_-adrenergic stimulation.

To demonstrate that the Ca^2+^ transients were representative of cardiac function, we next investigated the effect of β_1_-adrenergic stimulation upon cell shortening. Figure 2A shows representative traces of cell shortening (expressed as percentage of resting cell length, RCL) in control and detubulated rat ventricular myocytes before (blue and red traces, respectively) and during perfusion with specific β_1_-adrenergic stimulation (cyan and pink traces, respectively). Mean data (Fig. 2B) show that specific β_1_-adrenergic stimulation induced a significant increase in the amplitude of the cell shortening in both cell types (15.61 ± 0.85% RCL, *n* = 22 control cells vs. 6.76 ± 0.66% RCL *n* = 13, detubulated cells, two-way ANOVA, *P* < 0.05). Similar to Ca^2+^ transient experiments described above, in the absence of β_1_-adrenergic stimulation, detubulation significantly reduced the amplitude of the cell shortening (6.80 ± 0.81% RCL in control cells vs. 1.61 ± 0.17% RCL in detubulated cells, two-way ANOVA, *P* < 0.05). The percentage increase in cell shortening induced by specific β_1_- adrenergic stimulation was significantly larger in detubulated cells compared to control myocytes (401 ± 92% in detubulated cells, vs. 196 ± 40% in control cells, one-way ANOVA, *P <* 0.05, Fig. 2C). Thus, these data functionally confirm that β_1_-adrenergic receptors are more concentrated at the surface membrane than in t-tubular membrane of rat ventricular myocytes.

### Effect of β_2_-adrenergic stimulation on cardiac function

**Figure 3:**
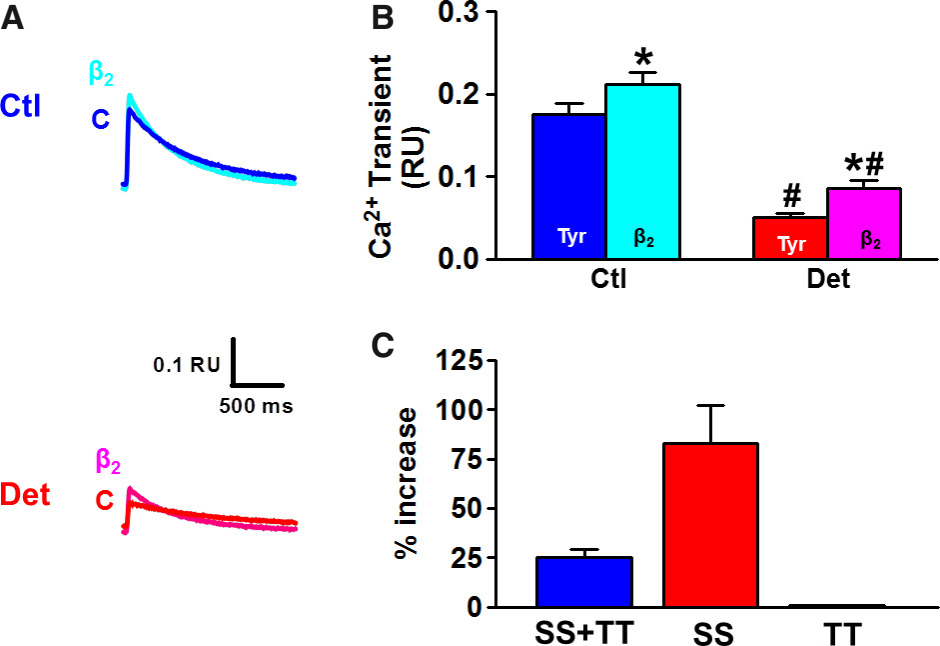
Effect of selective β_2_-adrenergic stimulation on Ca^2+^ transient of control and detubulated rat ventricular myocytes. (A) Ca^2+^ transient in representative control (Ctl) and detubulated (Det) myocytes before (blue and red traces) and after (cyan and pink traces) selective β_2_-adrenergic stimulation. (B) Mean ± SEM of Ca^2+^ transient amplitude under control condition (Tyr) and after β_2_-adrenergic stimulation (β_2_). Data are from 41 control and 20 detubulated cells. (C) Mean ± SEM of the percentage increase in the Ca^2+^ transient after β_2_-stimulation in control myocytes (surface sarcolemma + t-tubules; SS + TT) and detubulated myocytes (SS, surface sarcolemma). From these values, we calculated the percentage of increase at the TT (see text for details). #*P* < 0.05 between control and detubulated cells; *P < 0.05 between Tyrode and β_2_-adrenergic stimulation.

**Figure 4:**
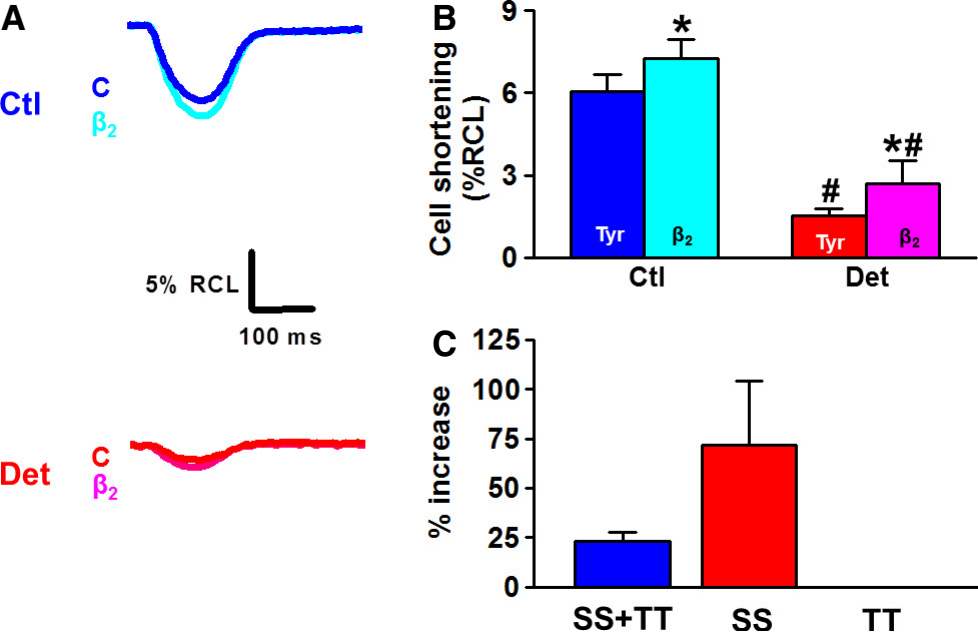
Effect of selective β_2_-adrenergic stimulation on cell shortening of control and detubulated rat ventricular myocytes. (A) Cell shortening in representative control (Ctl) and detubulated (Det) myocytes before (blue and red traces) and after (cyan and pink traces) selective β_2_-adrenergic stimulation. (B) Mean ± SEM of cell shortening expressed as a percentage of resting cell length (RCL) under control condition (Tyr) and after β_2_-adrenergic stimulation (β_2_). Data are from 23 control and 11 detubulated cells. (C) Mean ± SEM of the percentage increase in cell shortening after β_2_-stimulation in control myocytes (surface sarcolemma + t-tubules; SS + TT) and detubulated myocytes (SS, surface sarcolemma). From these values, we have calculated the percentage of increase at the t-tubules (TT, see text for details). #*P* < 0.05 between control and detubulated cells; *P < 0.05 between Tyrode and β_2_-adrenergic stimulation.

Figure 3A shows representative Ca^2+^ transients recorded from control (top) and detubulated (bottom) rat ventricular myocytes before (blue and red traces, respectively) and during perfusion with specific β_2_-adrenergic stimulation (10 μmol/L salbutamol and 1 μmol/L atenolol; cyan and pink traces, respectively). The mean data summarized in Figure 3B show that specific β_2_-adrenergic stimulation caused a significant increase in the amplitude of the Ca^2+^ transient in both cell types, albeit smaller than β_1_-adrenergic stimulation (0.212 ± 0.014 RU *n* = 41 control cells vs. 0.086 ± 0.009 RU *n* = 20 detubulated cells, two-way ANOVA, *P* < 0.05), thus confirming that β_1_-adrenergic stimulation is the major β-adrenergic receptor subtype in ventricular myocytes (see Xiang 2011 for review). In addition, the time to decay to 50% of Ca transient was not significantly changed by β_2_-adrenergic stimulation (382 ± 20 msec vs. 370 ± 22 msec in control cells and 800 ± 56 msec vs. 774 ± 64 msec in detubulated cells, two-way ANOVA, *P >* 0.05). The percentage increase in Ca^2+^ transient amplitude upon β_2_-adrenergic stimulation was significantly greater in detubulated than in control cells (83 ± 19% vs. 25 ± 4%, respectively, one-way ANOVA, *P* < 0.05, Fig. 3C). From these values we calculated the increase in Ca^2+^ transient amplitude at the t-tubules as for β_1_-adrenergic stimulation. We estimated that the percentage increase in Ca^2+^ transient at the t-tubules membrane was only 0.8%, suggesting that β_2_-adrenergic stimulation is almost exclusively due to surface sarcolemma receptors (∼100 times more efficient at surface sarcolemma than at t-tubules, Table 1). This also indicates that the increase in Ca^2+^ transient is due to 97% by β_2_-adrenergic stimulation at surface sarcolemma and only 3% at t-tubules (Table 1). Cell shortening was also assessed. Figure 4A shows representative traces of cell shortening in control and detubulated rat ventricular myocytes before (blue and red traces, respectively) and during perfusion with specific β_2_-adrenergic stimulation (cyan and pink traces, respectively). Mean data are summarized in Figure 4B and show that specific β_2_-adrenergic stimulation induced a significant increase in the amplitude of the cell shortening in both cell types, although of smaller amplitude than with β_1_-adrenergic stimulation (7.26 ± 0.70% RCL *n* = 23 control cells vs. 2.70 ± 0.82% RCL *n* = 11 detubulated cells, two-way ANOVA, *P* < 0.05). In agreement with Ca^2+^ transient experiments, quantitative results indicate that cell shortening upon β_2_-adrenergic stimulation was greater in detubulated than in control cells (71 ± 32% vs. 23 ± 5%, respectively, one-way ANOVA, *P* < 0.05, Fig. 3C) and that the increase in cell shortening at t-tubules membrane was 0.1%, hence confirming that β_2_-adrenergic stimulation appears to be not functional at the t-tubules. Thus, in agreement with the Ca^2+^ transient experiments, these data confirm that β_2_-adrenergic receptors are only functionally present at the surface sarcolemma of cardiac ventricular myocytes.

## Discussion

### Experimental approach

This study provides the first quantification of the functional subcellular localization of the two main cardiac β-adrenergic receptors: β_1_ and β_2_. Previous attempts to determine the localization of those receptors in cardiac myocytes have been mainly performed using immunocytochemistry (see Introduction), yet interpretation and quantification from such experiments can be challenging because antibody binding may depend on epitope accessibility rather than protein distribution itself. In contrast, the detubulation method we applied in this study allows a functional mapping of the proteins within the cell such that it is possible to discriminate the β-adrenergic response between the surface sarcolemma and the t-tubules membrane of ventricular myocytes. This method has been previously validated in cardiac myocytes lacking t-tubules (rat atrial cells) and it was shown to have no effect on proteins function (Brette et al. 2002). Importantly, formamide application does not alter β-adrenergic stimulation in rat atrial myocytes (Brette et al. 2002), strongly indicating that the experimental approach used in this study does not alter the proteins involved in the β-adrenergic intracellular pathway. Also, we have chosen the concentration of specific β-adrenergic stimulation (β_1_ and β_2_) to be in the mid-range response, based on previous studies (Balijepalli et al. 2006; Calaghan and White 2006). We believe that the physiological response observed here is more representative than when using maximal concentration of agonists. Thus, in this study, we used Ca^2+^ transient and cell shortening as physiological probes and compared the effects of specific β_1_- and β_2_-adrenergic stimulation in intact ventricular cells and in myocytes depleted from t-tubules (detubulated).

As classically described, the Ca^2+^ transient amplitude was significantly enhanced by adrenergic stimulation, albeit of smaller magnitude with β_2_- compared to β_1_-adrenergic stimulation (Bers 2002). We observed a greater response in detubulated myocytes that was irrespective of the type of adrenergic receptors activated. Our mathematical calculation indicates that functional β_1_-adrenergic receptors are more concentrated at the surface sarcolemma than at the t-tubules membrane while functional β_2_-adrenergic receptors are only present at surface sarcolemma of rat ventricular myocytes (Table 1).

The measure of the change in cell length (an index of contraction) induced by selective β_1_- and β_2_-adrenergic receptor stimulation on intact and detubulated rat ventricular myocytes is in agreement with Ca^2+^ transient measurements. We observed similar qualitative response in control and detubulated myocytes with both β-adrenergic stimulations, that is, β_1_-adrenergic stimulation is more efficient at the surface sarcolemma than at the t-tubules membrane whereas β_2_-adrenergic stimulation is mainly effective at the surface membrane of the cell.

### Relation to previous studies

Our functional distribution of β_1_-adrenergic receptors is in agreement with a previous study, which using a biochemical approach (radioligand binding) has shown that β_1_-adrenergic receptors are more abundant at surface sarcolemma than t-tubules (∼2 times) (He et al. 2005). However, this slightly contrasts with a study that used a biophysical approach (FRET measurement of cAMP) where it was found that cAMP increased to the same level at surface membrane and t-tubules (Nikolaev et al. 2010). It is not obvious why such discrepancy is present. It may be related to the fact that not all cAMP can activate downstream pathway (Steinberg and Brunton 2001). In addition, it is well known that observation of pathway activation (cAMP production) is not a reliable indicator of the functional response, for example Ca^2+^ transient (Hohl and Li 1991) because of local environment and accessory proteins such as phosphodiesterases and/or A kinase-anchoring proteins (Xiang 2011).

The distribution of β_2_-adrenergic receptors is more controversial. Biochemical studies have shown that β_2_-adrenergic receptors (i) have the same abundance at surface sarcolemma and t-tubules, using radioligand binding (He et al. 2005), (ii) are expressed at the surface sarcolemma and in the t-tubules of rat ventricular myocytes, using immunostaining (Zhou et al. 2000; Smyrnias et al. 2010). Our distribution of β_2_-adrenergic receptors reveals that these receptors are only functional at the surface membrane. It is possible that β_2_-adrenergic receptors are present at the t-tubules but not functional (because of local environment, above) or that these receptors are constitutively activated, and able to activate adenylyl cyclase in the absence of agonist (Zhou et al. 2000). In support of this hypothesis, Orchard's group has shown in two recent studies that there is a tonic PKA activation at the t-tubules (Chase et al. 2010; Chase and Orchard 2011), strongly suggesting that β_2_-adrenergic receptors are present and functional at the t-tubules and may be constitutively activated. The functional distribution of β_2_-adrenergic receptors contrasts with the results obtained with FRET measurement, where cAMP increased only at t-tubules and not surface sarcolemma during specific β_2_-adrenergic stimulation (Nikolaev et al. 2010). It is possible that FRET probe failed to detect the cAMP signal because of inhomogeneous intracellular distribution of the probe within the cytosol (as in the case for the PKA-based FRET probe, e.g*.,* [Zaccolo and Pozzan 2002]).

### Study limitations

The experiments were performed at room temperature. Accordingly, we used lower stimulation frequency than in situ (<1 Hz compare to ∼5 Hz at 37**°**C for rat). We chose these conditions because cell viability and dye loading is prolonged at this temperature. In addition, they match almost all benchmark data used for comparison (e.g., Brette et al. 2004; Nikolaev et al. 2010; Smyrnias et al. 2010).

In this study, we have investigated the end-point of cardiac physiological response (i.e., calcium transient and cell contraction). We did not record intermediate steps of the β-adrenergic intracellular pathway (e.g., L-type Ca^2+^ current). It is possible that the response of L-type Ca^2+^ current would not be representative of the functional effect observed, because in cardiac myocytes, there is no linear relationship between the amplitude of Ca^2+^ current and the Ca^2+^ release by the SR during β-adrenergic stimulation and this observation is true both at the global and local level (Hussain and Orchard 1997; Zhou et al. 2009). Therefore, it is very possible that, as described for biophysical and biochemical studies (above), intermediate steps in the pathway may respond in a different manner than the physiological response.

Another limitation of this study is that detubulation of myocytes may be incomplete. However, we expect this effect to be small (Despa et al. 2003; Pasek et al. 2008) and given that the β-adrenergic response (β_1_ and β_2_) is larger in detubulated myocytes than in control, this would underestimate the response at surface sarcolemma and would not change the conclusions of this study.

To conclude, this study provides for the first time a functional mapping of β_1_- and β_2_-adrenergic receptors in rat ventricular myocytes. In addition, this study highlights the importance of using a physiological approach rather than biochemical and/or biophysical approaches when studying signal transduction in cardiac myocytes.

## References

[b1] Balijepalli RC, Foell JD, Hall DD, Hell JW, Kamp TJ (2006). Localization of cardiac L-type Ca2+ channels to a caveolar macromolecular signaling complex is required for β2-adrenergic regulation. Proc. Natl. Acad. Sci. USA.

[b2] Bers DM (2002). Cardiac excitation-contraction coupling. Nature.

[b3] Brette F, Orchard C (2003). T-tubule function in mammalian cardiac myocytes. Circ. Res.

[b4] Brette F, Orchard CH (2006). Density and sub-cellular distribution of cardiac and neuronal sodium channel isoforms in rat ventricular myocytes. Biochem. Biophys. Res. Commun.

[b5] Brette F, Komukai K, Orchard CH (2002). Validation of formamide as a detubulation agent in isolated rat cardiac cells. Am. J. Physiol. Heart Circ. Physiol.

[b6] Brette F, Rodriguez P, Komukai K, Colyer J, Orchard CH (2004). beta-adrenergic stimulation restores the Ca transient of ventricular myocytes lacking t-tubules. J. Mol. Cell. Cardiol.

[b7] Calaghan S, White E (2006). Caveolae modulate excitation-contraction coupling and beta2-adrenergic signalling in adult rat ventricular myocytes. Cardiovasc. Res.

[b8] Chase A, Orchard CH (2011). Ca efflux via the sarcolemmal Ca ATPase occurs only in the t-tubules of rat ventricular myocytes. J. Mol. Cell. Cardiol.

[b9] Chase A, Colyer J, Orchard CH (2010). Localised Ca channel phosphorylation modulates the distribution of L-type Ca current in cardiac myocytes. J. Mol. Cell. Cardiol.

[b10] Despa S, Brette F, Orchard CH, Bers DM (2003). Na/Ca exchange and Na/K-ATPase function are equally concentrated in transverse tubules of rat ventricular myocytes. Biophys. J.

[b11] Fischmeister R, Castro LR, bi-Gerges A, Rochais F, Jurevicius J, Leroy J (2006). Compartmentation of cyclic nucleotide signaling in the heart: the role of cyclic nucleotide phosphodiesterases. Circ. Res.

[b12] Harvey RD, Calaghan SC (2012). Caveolae create local signalling domains through their distinct protein content, lipid profile and morphology. J. Mol. Cell. Cardiol.

[b13] He JQ, Balijepalli RC, Haworth RA, Kamp TJ (2005). Crosstalk of beta-adrenergic receptor subtypes through Gi blunts beta-adrenergic stimulation of L-type Ca2+ channels in canine heart failure. Circ. Res.

[b14] Hohl CM, Li QA (1991). Compartmentation of cAMP in adult canine ventricular myocytes. Relation to single-cell free Ca2+ transients. Circ. Res.

[b15] Hussain M, Orchard CH (1997). Sarcoplasmic reticulum Ca2 + content, L-type Ca2+ current and the Ca2+ transient in rat myocytes during beta-adrenergic stimulation. J. Physiol.

[b16] Kuschel M, Zhou YY, Cheng H, Zhang SJ, Chen Y, Lakatta EG (1999). G(i) protein-mediated functional compartmentalization of cardiac beta(2)-adrenergic signaling. J. Biol. Chem.

[b17] Nikolaev VO, Moshkov A, Lyon AR, Miragoli M, Novak P, Paur H (2010). Beta2-adrenergic receptor redistribution in heart failure changes cAMP compartmentation. Science.

[b18] Pasek M, Brette F, Nelson A, Pearce C, Qaiser A, Christe G (2008). Quantification of t-tubule area and protein distribution in rat cardiac ventricular myocytes. Prog. Biophys. Mol. Biol.

[b19] Smyrnias I, Mair W, Harzheim D, Walker SA, Roderick HL, Bootman MD (2010). Comparison of the T-tubule system in adult rat ventricular and atrial myocytes, and its role in excitation-contraction coupling and inotropic stimulation. Cell Calcium.

[b20] Steadman BW, Moore KB, Spitzer KW, Bridge JH (1988). A video system for measuring motion in contracting heart cells -1. IEEE Trans. Biomed. Eng.

[b21] Steinberg SF, Brunton LL (2001). Compartmentation of G protein-coupled signaling pathways in cardiac myocytes. Annu. Rev. Pharmacol. Toxicol.

[b22] Trafford AW, Diaz ME, Negretti N, Eisner DA (1997). Enhanced Ca2+ current and decreased Ca2+ efflux restore sarcoplasmic reticulum Ca2+ content after depletion. Circ. Res.

[b23] Xiang YK (2011). Compartmentalization of beta-adrenergic signals in cardiomyocytes. Circ. Res.

[b24] Zaccolo M, Pozzan T (2002). Discrete microdomains with high concentration of cAMP in stimulated rat neonatal cardiac myocytes. Science.

[b25] Zhou YY, Yang D, Zhu WZ, Zhang SJ, Wang DJ, Rohrer DK (2000). Spontaneous activation of beta(2)- but not beta(1)-adrenoceptors expressed in cardiac myocytes from beta(1) beta(2) double knockout mice. Mol. Pharmacol.

[b26] Zhou P, Zhao YT, Guo YB, Xu SM, Bai SH, Lakatta EG (2009). Beta-adrenergic signaling accelerates and synchronizes cardiac ryanodine receptor response to a single L-type Ca2+ channel. Proc. Natl. Acad. Sci. USA.

